# Regulatory Fit Demonstrates That Prohibitive Voice Does Not Lead to Low Performance Evaluation

**DOI:** 10.3389/fpsyg.2020.581162

**Published:** 2020-11-26

**Authors:** Lu Yang

**Affiliations:** School of Economics and Management, University of Science and Technology Beijing, Beijing, China

**Keywords:** prohibitive voice, prevention regulatory focus, safety performance evaluation, regulatory fit, moderated model

## Abstract

Voice behavior, the extra-role behavior of employees based on their sense of responsibility, plays an important role in organizational development. Research shows that an employee’s voice can have a positive impact on both the quality of decision-making and organizational performance. This study explores the relationship between the prohibitive voice and employees’ safety performance based on the theory of regulatory fit. The study examined 372 employees and their leaders in the Ningxia Hui Autonomous Region of China through a questionnaire survey. A moderated model was constructed, and the SPSS-PROCESS was applied to analyze the data. The study results show that prevention regulatory focus fit strengthened the positive association between the prohibitive voice and safety performance evaluation. This study provides a new perspective in understanding leaders’ evaluation of the prohibitive voice and concludes that the prohibitive voice should be encouraged in organizations as it promotes greater adherence to safety measures and helps reduce organizational development risks.

## Introduction

Voice behavior plays an important role in organizational development, and research shows that an employee’s voice has a positive impact on both the quality of decision-making and organizational performance (Burris, 2012). Effective use of voice in the workplace can enhance employees’ performance and contribute to their career advancement. In recent years, the relationship between voice and employee performance has been extensively studied (Rees et al., 2013; Chou and Barron, 2016). Some studies show that voice positively affects employee performance (Bryson et al., 2006; Whiting et al., 2008; Ng and Feldman, 2012; Chen and Hou, 2016), while others draw the opposite conclusion (Seibert et al., 2001; Hung et al., 2012). Song et al. (2019) argue that these two different conclusions may stem from prior researchers ignoring the effects of different types of voice on employee performance. In response to this gap in the research, Song et al. (2019) discussed the respective relationships between two types of voice (i.e., promotive voice and prohibitive voice) and employee performance. It may be helpful to note that the practice of thinking and talking about future developments and goals at work generally nurtures workplace enthusiasm (Kark and Van Dijk, 2007). For example, employees may excite managers by proposing new plans to improve their company’s market share; in turn, managers may view such employees as loyal (Burris, 2012), active (Liang et al., 2012), and deserving of higher performance evaluations. Meanwhile, employees may arouse negative emotions when they point out wrong behavior and potential hazards in their company (Carver and Scheier, 1998); in such situations, managers may not easily recognize these employees’ goodwill and, accordingly, may negatively evaluate their performance.

The term *voice* in this context refers to the extra-role behavior of employees based on their sense of responsibility, which can help leaders solve problems and improve organizational status. Employees may point out problems in the development of the organization and put forward suggestions and creative ideas to improve the current situation. Of course, voice behavior is a multidimensional construct, not a one-dimensional one, as demonstrated by previous scholars. For example, in the context of a speaker’s intention to improve organizational conditions, voice can be divided into *considerate voice* and *aggressive voice* (Hagedoorn et al., 1999). Meanwhile, based on employees’ voice behavior motivation, voice can be divided into *acquiescent voice*, *defensive voice*, and *prosocial voice* (Van Dyne et al., 2003). Additionally, Liu W. et al. (2010) divide voice into two types based on the target of the voice behavior: *speaking out* (voicing to peers) and *speaking up* (voicing to a supervisor). From the perspective of psychology, Liang et al. (2012) divide voice into *promotive voice* and *prohibitive voice* according to content; this approach is widely used in China, and has been validated by a broad range of scholars (e.g., Song et al., 2019). The current study adopts this method of analyzing voice and makes new contributions to the literature by focusing on the prohibitive voice. The differences between the promotive voice and prohibitive voice are summarized in Table 1.

**TABLE 1:** Comparison of promotive and prohibitive voices.

	**Promotive voice**	**Prohibitive voice**
Behavioral content	New ideas and suggestions to improve the organization’s organization’s operations. Future-oriented—take measures to make organization better in the future.	Problems and hidden dangers in the organization. Past or future oriented—point out harmful factors and abnormal behaviors.
Function	Provide advice on how the organization can better operate and develop. Apply team members’ unique knowledge to innovative practices.	Point out the factors that harm organizational development and draw collective attention to abnormal or dangerous behaviors of the team.
Appraisal	The speaker’s good intentions are easier to recognize, and leader’s leader’s evaluation tends to be more positive.	The speaker’s good intentions are not easily recognized and can lead to negative emotions and defensive behaviors. Leaders tend to give the speaker a less positive evaluation.
Emotions	Evokes positive emotions (e.g., optimism and enthusiasm).	Evokes negative emotions (e.g., anxiety and worry).
Strategies and goals	Eager approach to strategies to achieve success in meeting challenging goals.	Use of avoidance strategies to achieve security-related goals.

First, the promotive voice refers to the new ideas and innovative suggestions put forward by individuals to improve the current organizational situation and its future development; meanwhile, the prohibitive voice refers to the identification and expression of potential problems and existing dangers to avoid organizational failure. Employees using promotive voice often have their goodwill recognized more easily; they are considered more loyal (Burris, 2012), active, and positive (Liang et al., 2012) and, as noted above, are likely to receive more positive performance evaluations (Huang et al., 2018). On the other hand, employees using a prohibitive voice are less likely to have their goodwill recognized and therefore their voice behavior may be perceived negatively. While the prohibitive voice can function to halt dangerous behaviors and reduce organizational loss in time; thus, it can solve current problems and is more effective than promotive voice (Liang et al., 2012). Additionally, the prohibitive voice can improve team safety performance gains (Li et al., 2017) and promote team innovation through team reflection (Liang et al., 2019). Therefore, the prohibitive voice is indispensable to a team or organization (Liang et al., 2012). However, because the prohibitive voice is often used to point out existing problems and errors, the speakers’ intended helpfulness is generally not easily recognized and may yield negative emotions and defensive behaviors among leaders and co-workers (Van Dyne et al., 1995; Liang et al., 2012; Chamberlin et al., 2017). Accordingly, the prohibitive voice is more likely to emotionally exhaust speakers than the promotive voice (Sessions et al., 2019); thus, employees tend to avoid using a prohibitive voice. As noted above, prior studies conclude that speakers using the prohibitive voice usually receive negative evaluations and face negative social consequences (Liang et al., 2012). However, this is not always the case: prohibitive voice speakers can also receive positive appraisals. To bridge the research gap and encourage the effective use of the prohibitive voice, this study seeks to uncover the circumstances under which prohibitive speakers may receive favorable performance evaluations. In particular, the present study focuses on performance—namely, safety performance and task performance—and seeks to locate the positive effect of the prohibitive voice on safety performance evaluations.

Present study findings indicate that utilizing the prohibitive voice does not usually lead to positive safety performance evaluation, indicating the presence of some factors that moderate the appraisal process. To be sure, leaders and employees have different goals and different means to achieve their goals. The tendency to move toward some type of target state is defined as *regulatory focus* (Higgins, 1997). Higgins (1997) distinguishes two kinds of self-regulatory modes: *promotion regulatory focus* and *prevention regulatory focus*. As described in more detail below, promotion regulatory focus centers on the ideal-self, and satisfies the needs of individual growth, expectations, and aspirations, while prevention regulatory focus centers on an ought-self that satisfies the needs of individual safety, responsibility, and obligation. According to regulatory fit theory, people with different regulatory foci react differently to the same situations (Higgins, 1997) and people generally focus on the information that fits their individual regulatory focus (Higgins, 2000). Hence, when employees have a high regulatory fit with their supervisors, their focus is consistent. Notably, prevention-focused leaders and employees are often concerned with safety goals. When there is a strong prevention focus fit between employees and their supervisors, the positive relationship between prohibitive voice and safety performance evaluation is strengthened. Thus, the regulatory fit will influence leaders’ evaluation of prohibitive speakers. As such, regulatory fit theory is key to understanding a leader’s attitude to the prohibitive voice. By integrating regulatory fit theory, this study further explores the moderating effect of prevention focus fit and explicates the influence mechanism of the prohibitive voice on safety performance evaluation.

Work performance is classified into two categories: *task performance* and *contextual performance* (Borman and Motowidlo, 1993). Meanwhile, as an independent category, *safety performance* is an important form of work performance, especially for high-risk industries and information systems. More specifically, safety performance signifies the actions or behaviors that individuals exhibit in almost all jobs to promote the health and safety of workers, clients, the public, and the environment (Burke et al., 2002). Notably, some researchers offer other definitions of safety performance. For example, Liu S. X. et al. (2010) define safety performance as the occurrence of safety accidents and their consequences (Liu S. X. et al., 2010). Moreover, safety performance has also been mobilized to signal the ability of enterprises to control and react when accidents occur (Kjellén and Albrechtsen, 2017). Christian et al. (2009) states that safety performance includes both safety performance behaviors and safety outcomes. The former refers to the safety behavior of employees, while the latter refers to accidents, injuries, and other tangible events and results. The current study employs Burke et al. (2002) definition of safety performance because it is widely accepted. As noted above, the prohibitive voice points out existing problems and wrongful actions in an organization, and this draws extensive attention to abnormal or dangerous behaviors. If acted upon correctly, staff will cease such behaviors and supervisors will cease and withdraw hazardous plans. As a result, organizations will reduce loss and improve safety performance. To facilitate such work, Griffin and Neal (2000) developed a scale to measure employee safety performance. Sample items from the scale include: “I help my co-workers when they are working under risky or hazardous conditions,” “I scan the environment for unsafe actions by the team,” and “I voluntarily carry out tasks or activities that help improve workplace safety.” In this way, prohibitive voice points out harmful factors in the organization and functions to immediately stop dangerous and irregular behaviors. Therefore, prohibitive speakers may receive high safety performance evaluations. Thus, the following hypothesis is presented:

Hypothesis 1: The prohibitive voice is positively related to employee safety performance evaluation.

As touched upon above, Higgins (1997) proposed regulatory focus theory, an expansion of self-discrepancy theory, and distinguished between promotion regulatory focus and prevention regulatory focus. Together with regulatory fit theory, regulatory focus theory forms the system for self-regulation theory. Self-regulation is a process whereby individuals strive to change or control their thoughts and reactions to achieve specific goals (Geers et al., 2005). According to a person’s regulatory focus, promotion-focused self-regulation reflects the need for individual growth, improvement, and development (Tumasjan and Braun, 2012); people with a promotion focus eagerly use active strategies to achieve positive outcomes (Levontin et al., 2004), and position themselves as an ideal-self (Park et al., 2017). In contrast, prevention-focused self-regulation reflects the need for individual safety and stabilization (Tumasjan and Braun, 2012); accordingly, people with prevention focus use vigilant and avoidance strategies to avoid negative outcomes (Levontin et al., 2004) and position themselves as an ought-self (Park et al., 2017). Prevention focus is thus concerned with duties, responsibilities, safety, and security (Levontin et al., 2004).

People experience a regulatory fit when they use goal pursuit means that fit their regulatory orientation and pay close attention to the information that fits their regulatory focus (Higgins, 2000). For example, when people are promotion-focused, they are sensitive to creative ideas and novel thoughts (Levontin et al., 2004); meanwhile, prevention-focused individuals are more sensitive to safety, security, and ought issues (Levontin et al., 2004). Important to note here is that regulatory fit increases the value of what people are doing—when a regulatory fit is strong, people are more motivated. Previous studies have argued that the prohibitive voice proposes corrective measures to stop actions that conflict with a leader’s ideals (Burris, 2012; Liang et al., 2012). Researchers have also found that regulatory focus plays a pivotal role in social interactions between leaders and employees (De Cremer et al., 2009). Prevention-focused people aim to realize safety and stability–key foci of prohibitive voice. Moreover, when employees and their leader have high prevention regulatory fit, they are both more concerned with prohibitive voice; notably, this relation ultimately affects the relationship between prohibitive voice and safety performance evaluations. Along these lines, the present study also seeks to explore the moderating effect of prevention regulatory fit on the relation between the prohibitive voice and performance evaluation.

Helpful to note here is that people with different regulatory foci pay attention to different types of information. Regulatory fit increases the value of what people do. Promotion-focused people are more sensitive to positive information, such as that related to advancement and creativity, while prevention-focused individuals are more sensitive to negative information, such as that related to loss. If leaders have a prevention focus, they tend to achieve security-related goals and expect to avoid adverse risks. Prevention-focused employees point out risks and problems and warn leaders and colleagues about harmful behaviors, meeting the leader’s expectations, and improving the safety performance of the organization. In this way they share a high prevention regulatory fit and common concerns, so that supervisors are interested in the content of the voice presented and deliver high evaluations to prohibitive speakers. Therefore, when a high prevention regulatory fit is present, leaders will give a high-performance evaluation to employees who utilize the prohibitive voice. In contrast, if the leader-employee prevention regulatory fit is low, both parties have different foci and the leader will not be interested in the content of the prohibitive voice. Accordingly, we derive the following hypothesis:

Hypothesis 2: Prevention regulatory focus fit will strengthen the positive relationship between prohibitive voice and employee safety performance evaluation. Specifically, the relationship is stronger when the leader-employee prevention regulatory fit is high than when it is low.

**FIGURE 1:**
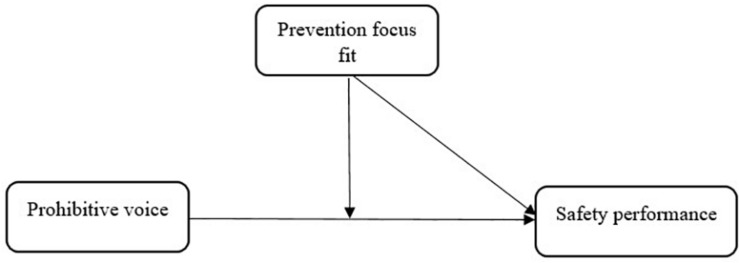
Conceptual model.

To test the two hypotheses, we developed the moderated model depicted in Figure 1.

In sum, this study seeks to uncover the relationship between voice and safety performance evaluations. Prior research evidences that prohibitive voice points out the problems in an organization and often triggers negative emotions. Moreover, existing work has shown a general negative relationship between prohibitive voice and employee performance (Klaas and DeNisi, 1989; Liang et al., 2012; Chamberlin et al., 2017). To present information that can be used to help staff overcome concerns and encourage their use of prohibitive voice, we use empirical data to clarify the positive effects that may be experienced by employees who use the prohibitive voice and demonstrate that it can lead to high safety performance evaluations. By integrating regulatory fit theory, we further study the relationship between prohibitive voice and safety performance evaluations. The following section describes our survey procedure and data analysis. Section three provides the research results, where we present the discriminate validity of the variables, descriptive statistics, and hypothesis testing. This is followed by Discussion section and then the Conclusion.

## Materials and Methods

### Participants and Procedure

Data were collected from a provincial bank in the Ningxia Hui Autonomous Region in China. Research participants consisted of 405 employees of various ages from 45 branch networks who accepted our survey. The questionnaire was not anonymous and was instead designed to respond to the unique qualities of the employees and their leaders. One author of this study was on-site to distribute and collect the questionnaires. Ultimately, we retained 372 paired samples after deleting the questionnaire with incomplete information, yielding a 92% overall response rate. Demographic data were provided by the human resources department of the bank before the formal survey was distributed. Questionnaires were distributed based on the company’s male to female ratio. All respondents were informed that their participation was voluntary and their information would be treated with strict confidentiality.

The 372 employees were separated into 45 work groups, each with an average of 8.27 individuals. Among them, 79.6% of participants were female. The average age of participants was 29.87 years (SD = 7.11). Educational level was divided into three categories: college degree (12.2%), bachelor’s degree (87.0%), and master’s degree (0.8%). The size of the groups ranged from 4 to 21, which fits the standard of working groups set by Jackson and Joshi (2011) of between 3 and 50 participants.

### Ethics Statement

The study was reviewed and approved by the ethical committee of the University of Science and Technology Beijing. Written informed consent was obtained from all employees and their managers. All participants were informed of their right to withdraw from the survey at any time.

### Measures

All measurement items were used in prior studies and were originally drafted in English. The items were then back translated to ensure accuracy (Brislin, 1980). Our analysis produced three factors: prohibitive voice, prevention focus fit, and employee safety performance. Employees reported their prohibitive voice. Employees and their leaders completed a survey of their prevention regulatory focus. Supervisors rated the employees’ safety performance.

#### Control Variables

The demographic variables included age, gender (1 = male, 2 = female), tenure, and educational attainment (1 = college degree, 2 = bachelor’s degree, 3 = master’s degree). The demographic variables of the employees (i.e., age,gender,tenure and educational level) were assigned as the control variables. Specifically, age and tenure are related to one’s accumulated work experience and thus influence performance (Shirom et al., 2008). As men generally receive better performance evaluations from their supervisors than women (Watkins et al., 2006), we also controlled for gender. As employees’ educational level is linked with profession, and supervisors give better appraisals to employees with higher educational attainment, educational attainment was also controlled.

#### The Prohibitive Voice

The research team measured the prohibitive voice using the three-item scales adapted from Liang et al. (2012) five-item scales. Employees rated the extent to which each statement described them via a 7-point response scale ranging from 1 = not at all true of me to 7 = very true of me. Sample items include: “I often point out the inappropriate behavior that affects team performance,” “I often point out the deficiencies and hidden dangers in the process of business handling,” and “I often point out the problems and shortcomings of the team” (α = 0.908).

#### Prevention Regulatory Fit

We assessed self-regulatory focus using the General Regulatory Focus Measure (GRFM) developed by Lockwood et al. (2002). The GRFM has primarily been used in applied research that views self-regulatory focus as a relatively stable motivation (i.e., trait approach). This scale contains two sub-scales with 9 items each. One sub-scale assesses promotion focus (e.g., “I frequently imagine how I will achieve my hopes and aspirations”; α = 0.861 for leaders and 0.909 for employees), and the other measures prevention focus (e.g., “In general, I am focused on preventing negative events in my life and work”; α = 0.714 for leaders and 0.844 for employees). We used a 7-point rating scale (1 = not at all true of me to 7 = very true of me) and chose the absolute value of difference between employees’ and leaders’ prevention regulatory focus as regulatory non-fit. There was no common variance.

#### Safety Performance

Consistent with Mehra et al. (2002), each participant was assessed by their direct supervisor. The safety performance items were drawn from Burke et al. (2002) general safety performance measure (“The employee handles business prudently and prevents and controls risks actively,” “The employee follows the regulations strictly and has no operational errors,” and “The employee pays attention to all kinds of information and proactively discovers potential risks.”). The safety performance items were based on the role responsibilities required by the company. The scales with 7-point answering formats (1 = never to 7 = constantly) were internally consistent (α = 0.876).

### Data Analysis

We initially used MPLUS to examine the discriminant validity of the key variables in this study. The adequacy of the model was tested based on the χ2/df, TLI, CFI, GFI, NFI, IFI, and RMSEA. We subsequently presented the means, standard deviations, and correlations among the study variables. Following the two preliminary data analyses, we used SPSS-PROCESS to analyze the moderated model.

## Results

### Discriminant Validity of Variables

Prior to testing the hypotheses, confirmatory factor analyses (CFA) was conducted using MPLUS to rate the discriminant validity of the survey measures. The CFA results are provided in Table 2. The three-factor model (i.e., prohibitive voice, prevention focus non-fit, and safety performance) fit the data well. We next combined prohibitive voice and safety performance into one variable to form a two-factor model. Finally, we combined prohibitive voice, prevention focus non-fit, and safety performance into a one-factor model, and the results showed the model fit the data poorly, indicating that the three constructs have good discriminate validity.

**TABLE 2:** CFA results.

	**χ^2^**	**df**	**CFI**	**TLI**	**RMSEA**	**SRMR**
M1——Three factors	403.866	87	0.863	0.835	0.098	0.075
M2——Two factors	866.358	89	0.665	0.605	0.152	0.116
M3——One factor	1413.865	90	0.429	0.334	0.197	0.154

### Descriptive Statistics

Table 3 presents the descriptive statistics and correlations of the study constructs. An inspection of the correlations reveals that employees’ prohibitive voices are not significantly related to their age, gender, educational attainment, or tenure. Moreover, employee safety performance is also not significantly related to their age, gender, educational attainment, or tenure. Consistent with Hypothesis 2, the prohibitive voice positively relates to safety performance and the effect is significant (*r* = 0.173, *p* = 0.001). Prevention focus non-fit negatively relates to safety performance and the effect is significant (*r* = −0.124, *p* = 0.017), indicating that prevention focus fit affects safety performance evaluation positively.

**TABLE 3:** Means, standard deviations, and correlations of variables.

	**Mean**	**SD**	**1**	**2**	**3**	**4**	**5**	**6**	**7**
(1) Age	29.99	7.25	1						
(2) Gender	1.80	0.40	0.30	1					
(3) Educational attainment	1.89	0.34	−0.556***	−0.013	1				
(4) Tenure	3.10	4.04	0.477	−0.007	−0.294***	1			
(5) Prohibitive voice	5.27	0.91	0.014	−0.020	0.025	0.035	1		
(6) Prevention focus non-fit	1.34	1.33	−0.083	−0.050	0.066	−0.040	0.092	1	
(7) Safety performance	5.67	1.07	0.014	0.019	0.030	−0.082	0.173**	−0.124*	1

N = =372. *p < 0.05; **p < 0.01; ***p < 0.001.

### Hypothesis Testing

**FIGURE 2:**
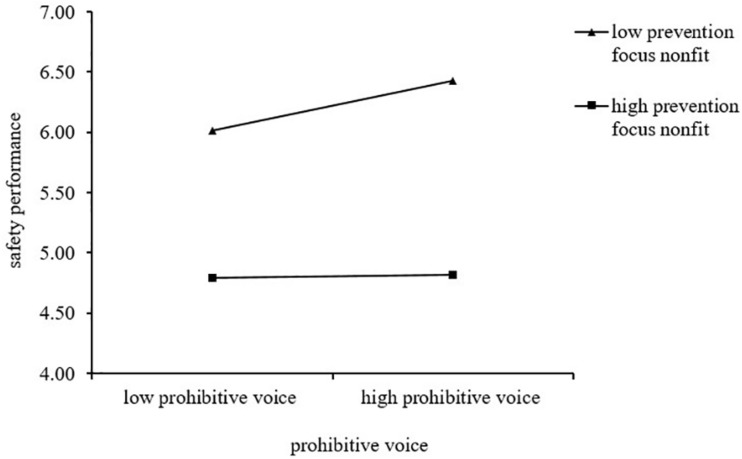
The interactive effect of the prohibitive voice and prevention focus fit on safety performance.

Hypothesis 1 predicted the positive relationship between the prohibitive voice and employee safety performance evaluation. To test the hypothesis, we incorporated the prohibitive voice into the model. The coefficient of the prohibitive voice and safety performance was significant and positive (β = 0.2283, *p* < 0.001), indicating that the prohibitive voice positively relates to employee safety performance evaluation. Therefore, Hypothesis 1 was supported. Hypothesis 2 predicted that prevention regulatory focus fit strengthened the positive relationship between the prohibitive voice and employee safety performance evaluation. We derived 95% confidence intervals (CI) to test our moderated hypothesis and research question. The coefficient of prevention focus non-fit and safety performance was significant and negative (β = −0.1094, *p* < 0.01), suggesting that prevention focus fit impacted safety performance evaluation positively. At lower levels of prevention focus non-fit (higher prevention focus fit), the effect of the prohibitive voice on safety performance evaluation was significant (effect = 0.3386 CI [0.1727, 0.5044]). Whereas at higher levels of prevention focus non-fit (lower prevention focus fit), the effect of the prohibitive voice on safety performance evaluation was not significant (effect = 0.1181 CI [−0.322, 0.2684]). The result indicates that when leader and employee have a high prevention focus fit, employees who utilize the prohibitive voice can achieve a high safety performance evaluation. Figure 2 shows the interaction effect of prohibitive voice and prevention focus fit on safety performance. The prohibitive voice is more strongly related to safety performance evaluation when employees have a higher prevention regulatory focus fit with their leaders. However, the positive effect of the prohibitive voice on safety performance evaluation is weaker and not significant when employees have a lower prevention regulatory focus fit with their leaders. Hence, Hypothesis 2 was also supported.

## Discussion

Reviews of recent literature highlighted different types of voice effects on employee performance evaluations (Li et al., 2017; Song et al., 2019). As noted in the Introduction, employees using the prohibitive voice point out risks or harmful factors in the organization; they may be perceived as complaining or fault-finding and evoke leaders’ negative emotions (Van Dyne et al., 1995; Liang et al., 2012). Thus, prohibitive voice speakers are regarded as destroyers of sportsmanship (Liang et al., 2012) and tend to receive low performance evaluations (Klaas and DeNisi, 1989). Prior studies have focused on the negative effect of prohibitive voice on employee performance evaluation, but only offer partial insights. This study takes the category of performance—namely, safety performance and task performance. The findings reveal a positive association between the prohibitive voice and employee safety performance and demonstrate that prohibitive voice can lead to positive performance evaluations. Moreover, by integrating regulatory fit theory, we examined this relationship and explored the moderating effect of prevention focus fit. The findings show that the prohibitive voice strongly relates to safety performance evaluation when employees have a higher prevention regulatory focus fit with their leaders, whereas the positive relationship between prohibitive voice and safety performance evaluation is weaker when employees have a lower prevention regulatory focus fit with their leaders. Therefore, prevention regulatory fit moderates the relationship.

### Theoretical Implications

This study makes several theoretical contributions. First, most studies show that the prohibitive voice negatively affects employee performance evaluation (Van Dyne et al., 1995; Liang et al., 2012; Chamberlin et al., 2017; Huang et al., 2018). However, this study classified performance into safety performance and task performance and found that the prohibitive voice can lead to high safety performance evaluation. These results demonstrate that taking into account the type of performance is vital to understanding the effect of the prohibitive voice on individual performance. This study may provide a possible explanation for the differences in academic research regarding the relationship between voice and performance; moreover, it may also promote more research on the voice–employee performance relationship that focuses on voice types and performance.

Second, this study also has important implications for understanding the positive aspect of the prohibitive voice, especially for employees at the individual level. Empirical research on the outcomes of the prohibitive voice is mostly negative (Klaas and DeNisi, 1989; Liang et al., 2012; Chamberlin et al., 2017) because, as explained above, the prohibitive voice usually arouses negative emotions and defensive behaviors (Lin and Johnson, 2015). This study verified that the prohibitive voice can also lead to positive employee performance and that this effect relates to performance type and the leader’s regulatory focus. Our results suggest that an exploration of the positive effects of the prohibitive voice at different organizational levels may reveal more about this relationship. These findings enrich existing understandings of the prohibitive voice and may facilitate an improved understanding of the psychological processes that link the prohibitive voice to its consequences.

A third contribution of the study is that it yields theoretical insights into how the prohibitive voice influences employee performance by considering the prevention regulatory fit of leaders and employees. Regulatory focus fit theory provides a new perspective to better understand how leaders evaluate worker’s use of the prohibitive voice. Research shows that employees make better decisions at work when they have higher regulatory fit with their organizations (Higgins, 2000). Along these lines, our findings indicate that leaders give better performance evaluations to prohibitive speakers when they have a higher prevention regulatory fit because they share a common focus and pursuit. Therefore, prevention regulatory focus fit strengthens the positive relationship between the prohibitive voice and employees’ safety performance. The regulatory fit theoretical perspective can also offer new insights into the relationship between voice and other types of employee performance.

### Managerial Implications

This study also offers important practical insights. First, current research on supervisors’ evaluations of prohibitive speakers are generally negative. We examined the relationship between the prohibitive voice and employee safety performance and confirmed their positive relationship. At the individual level, this finding can help reduce staff fears when they wish to utilize the prohibitive voice. At the organizational level, it can encourage employees to utilize the prohibitive voice when faced with potential problems and dangers present, thereby changing the “silence is golden” workplace norm while also improving organizational safety performance.

Furthermore, this study confirms the importance of prevention regulatory focus fit in moderating the positive relationship between the prohibitive voice and employee safety performance. On the one hand, managers should not only focus on organizational growth, but also on safety-related goals; accordingly, they need staff to utilize both promotive and prohibitive voices. Generally, the promotive voice evokes positive emotions and is more welcome in organizations. It is therefore important for leaders to provide a good environment for utilizing the prohibitive voice. For example, managers can implement the practice of “necessary evils” (Molinsky and Margolis, 2005) and let employees speak up and utilize their prohibitive voice. On the other hand, when using the prohibitive voice, employees should consider supervisors’ regulatory foci to avoid low performance evaluations. When these actors have a high prevention regulatory fit, use of the prohibitive voice is more likely to result in a high safety performance rating by leaders. Knowing leaders’ preferences, especially in terms of their regulatory foci, helps employees protect themselves and receive high performance evaluations when they utilize prohibitive voice.

### Limitations and Suggestions for Future Research

While this study achieved valuable results, there are some limitations worth noting. First, the sample was confined to the financial industry, so the research findings are not widely representative and may not be generalizable to other fields. Different industries place emphasis on safety performance variably, and the collective regulatory foci in different companies also vary. Regulatory focus theory highlights that the team voice can act as a mechanism through which members pursue collective goals (Li et al., 2017). Therefore, further research should enlarge the sample size and include data from other industries.

Second, the particular stressors characteristic of the sample’s financial industry setting (Malamardi et al., 2015) may have also influenced the findings; however, this was not explored in this study. To be sure, bank employees may feel unique pressures; moreover, their quality of life is often negatively affected by occupational stress. For example, Kan and Yu (2016) studied depression among bank employees and found that extrinsic effort, overcommitment, and work-family conflict had positive effects on depressive symptoms. Because work-related stress is common in the banking sector, future studies will do well to pay attention to whether it influences the relationship between voice and employee performance (Giorgi et al., 2019).

Third, we measured regulatory focus and safety performance during the same time period. This is a cross-sectional study, and there were limitations in explaining causal relationships. Employees’ safety performance evaluations may change over time, and the relationship between prohibitive voice and performance is complicated. A longitudinal study design can better evaluate this complex relationship and report the changes.

Fourth, in line with prior studies (Liang et al., 2012; Song et al., 2019), we adopted subjective measures and asked participants to rate their prohibitive voices themselves. However, subjective measures are vulnerable to systematic bias (Bommer et al., 1995) and random error (Bollen and Paxton, 1998). Future studies should combine the self-rating scores with leader assessment scores so that the data is more objective and the study is more robust.

Finally, our data were collected in China, and cultural differences that may affect the generalizability of the findings have not been considered. To ensure external validity, future scholars should expand the sample to include other countries and conduct cross-national research.

## Conclusion

In conclusion, the present study finds a positive association between the prohibitive voice and employee safety performance evaluation; moreover, it also uncovers that prevention regulatory focus fit strengthens this relationship. That is, the relationship strengthens when high leader–employee prevention regulatory fit is present. These findings bridge a gap in existing research, affirm the use of prohibitive voice, and have important managerial and practical implications.

## Data Availability Statement

The raw data supporting the conclusion of this article will be made available by the authors, without undue reservation.

## Ethics Statement

The studies involving human participants were reviewed and approved by ethical committee of the University of Science and Technology Beijing. The patients/participants provided their written informed consent to participate in this study.

## Author Contributions

LY substantially contributed to all aspects of the research, including research concept and design, drafting the manuscript and data analysis. LY repeatedly revised and refined the content of the manuscript, and contributed to the final approval of the version to be published.

## Conflict of Interest

The authors declare that the research was conducted in the absence of any commercial or financial relationships that could be construed as a potential conflict of interest.
